# Histopathologic evidence of intimal hyperplasia in carotid artery webs associated with stroke

**DOI:** 10.17879/freeneuropathology-2025-7139

**Published:** 2025-08-26

**Authors:** Farhan Khan, Alisa Nobee, Skylar Lewis, John E. Donahue, Shadi Yaghi

**Affiliations:** 1 Department of Neurology, Brown University, Providence, RI 02903, USA; 2 Department of Pathology, Brown University Providence, RI 02903, USA

**Keywords:** Carotid artery web, Stroke, Intimal hyperplasia

## Abstract

**Background:** Carotid artery webs (CWs) are an underrecognized cause of ischemic stroke, particularly in younger patients who lack conventional vascular risk factors. CWs are thought to represent an intimal variant of fibromuscular dysplasia (FMD); however, histopathologic data supporting this hypothesis remain limited. We report a case series of three patients with CW-related ischemic stroke who underwent carotid endarterectomy (CEA), allowing for histological analysis of the resected specimens.

**Methods:** We retrospectively reviewed patients admitted to a Comprehensive Stroke Center between January 2015 and April 2025 with ischemic stroke or transient ischemic attack attributed to an ipsilateral carotid web who subsequently underwent carotid endarterectomy. Clinical data, imaging findings, and histopathologic features were analyzed. All cases met criteria for embolic stroke of undetermined source (ESUS) prior to surgery.

**Results:** Three patients with CW-related stroke underwent carotid endarterectomy following recurrent events or high embolic risk. In two cases, superimposed thrombi led to initial misdiagnoses such as soft plaque or dissection. Histopathologic analysis consistently demonstrated fibrovascular tissue with intimal fibroid hyperplasia and myxoid degeneration, without lipid-rich plaques or inflammatory infiltrates. No patients experienced recurrent stroke or TIA by the time of their last documented follow-up.

**Conclusions:** CWs represent a distinct non-atherosclerotic pathology characterized by intimal hyperplasia and myxoid degeneration. Superimposed thrombus may complicate diagnosis, often mimicking plaque or dissection. Advanced imaging, including MR vessel wall imaging and intravascular optical coherence tomography (OCT), can aid in accurate identification. Carotid revascularization may be effective in selected patients, particularly those with recurrence or ESUS. Prospective studies are needed to inform standardized diagnostic and therapeutic strategies.

## Introduction

Carotid artery web (CW) is an underrecognized cause of ischemic stroke^1^. CWs appear as shelf-like projections arising from the posterior wall of the proximal internal carotid artery^3^. CWs have a reported prevalence of 1–3 % among patients with large vessel occlusion and is more commonly observed in Black women^2^. Hemodynamic studies suggest that this morphology leads to increased recirculation time and high wall shear stress, which promotes blood stasis and endothelial injury, thereby facilitating thrombogenesis^4^. Because of their subtle appearance on imaging, CWs are frequently overlooked, resulting in delayed or missed diagnoses^5^.

Despite medical treatment, CWs are associated with a high rate of recurrent stroke^2,5,6^. These recurrence rates are higher than those reported in large randomized controlled trials (RCTs) studying embolic stroke of undetermined source (ESUS), supporting the consideration of more aggressive interventions, such as carotid revascularization, for secondary prevention^7,8^. A recent meta-analysis reported recurrence rates as high as 32 % in patients with ipsilateral CWs and found no significant difference in efficacy between carotid stenting and endarterectomy^9^.

Prior literature suggests that CWs may represent a variant of fibromuscular dysplasia (FMD), involving intimal hyperplasia, in contrast to the more common medial hyperplasia seen in classical FMD. Due to limited data on the histopathology of carotid artery webs, we present a case series of patients who underwent carotid endarterectomy at our institution.

## Methods

### Clinical data

The data supporting the findings can be obtained from the corresponding author upon reasonable request. The study was approved by the local institutional review board, and informed consent was waived due to the nature of the study. This retrospective observational case series includes patients who developed clinical and neuroimaging evidence of ischemic stroke and underwent carotid endarterectomy. We only included patients admitted to the Comprehensive Stroke Center with strokes occurring between January 2015 and April 2025, where the ischemic stroke was attributed to an ipsilateral carotid web. Carotid webs were identified by reviewing CT angiograms of the neck in multiple planes (axial, sagittal, and coronal) by treating vascular neurologists, neuroradiologists, and neurosurgeons. Carotid web revascularization was performed only when no alternative stroke etiology was identified, as these strokes were classified as ESUS (embolic stroke of undetermined etiology) as per TOAST criteria by board-certified vascular neurologists. Carotid revascularization was performed according to institutional protocols. Demographic data, clinical presentation, laboratory and radiologic results were recorded by approved personnel through chart review.

### Pathology

All surgical specimens obtained during carotid endarterectomy (CEA) were submitted for gross and histopathological examination by board-certified neuropathologists. Specimens were fixed in 10 % neutral-buffered formalin, processed, and paraffin-embedded using standard protocols. Tissue blocks were sectioned at 4–5 microns and stained with hematoxylin and eosin (H&E). Gross examination included an evaluation of specimen size, color, texture, and the presence of thrombus, calcification, or fibrous thickening. All slides were reviewed by at least one board-certified neuropathologist blinded to clinical imaging findings. Representative photomicrographs were captured at low (2×) and high (20×) magnification using a digital slide scanner.

## Results

### Case 1

**Figure 1:**
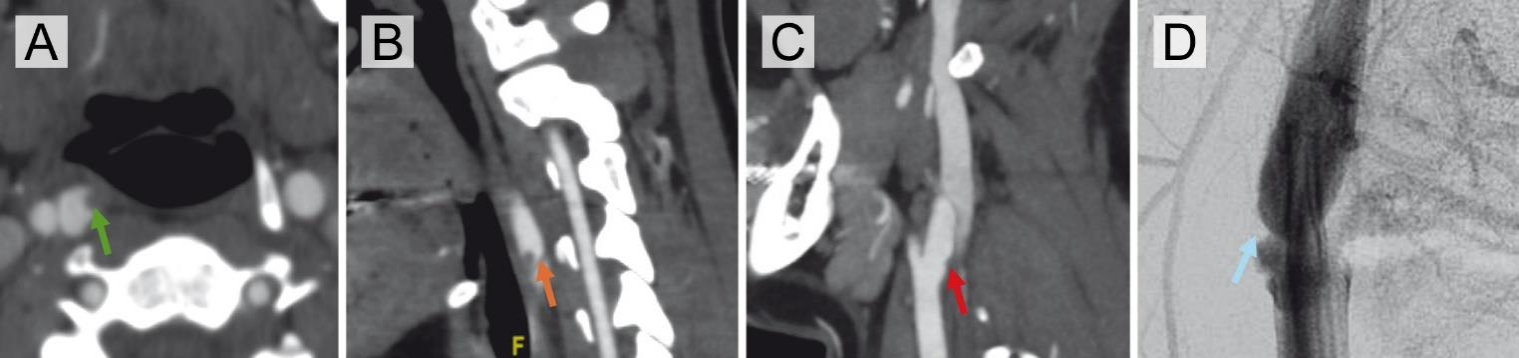
**Figure 1.** Imaging findings of a right carotid artery web with superimposed thrombus and interval resolution. **(A)** Axial CTA demonstrating a shelf-like projection along the posterior wall of the right internal carotid artery (ICA) with superimposed thrombus (green arrow). **(B)** Corresponding sagittal CTA confirming the presence of thrombus overlying a carotid web in the right ICA (orange arrow). **(C)** Follow-up sagittal CTA performed after anticoagulation shows resolution of thrombus with a small residual carotid web in the proximal right internal carotid artery (red arrow). **(D)** Digital subtraction angiography (DSA) confirms the presence of a subtle right carotid artery web without flow-limiting stenosis (blue arrow).

**Figure 2:**
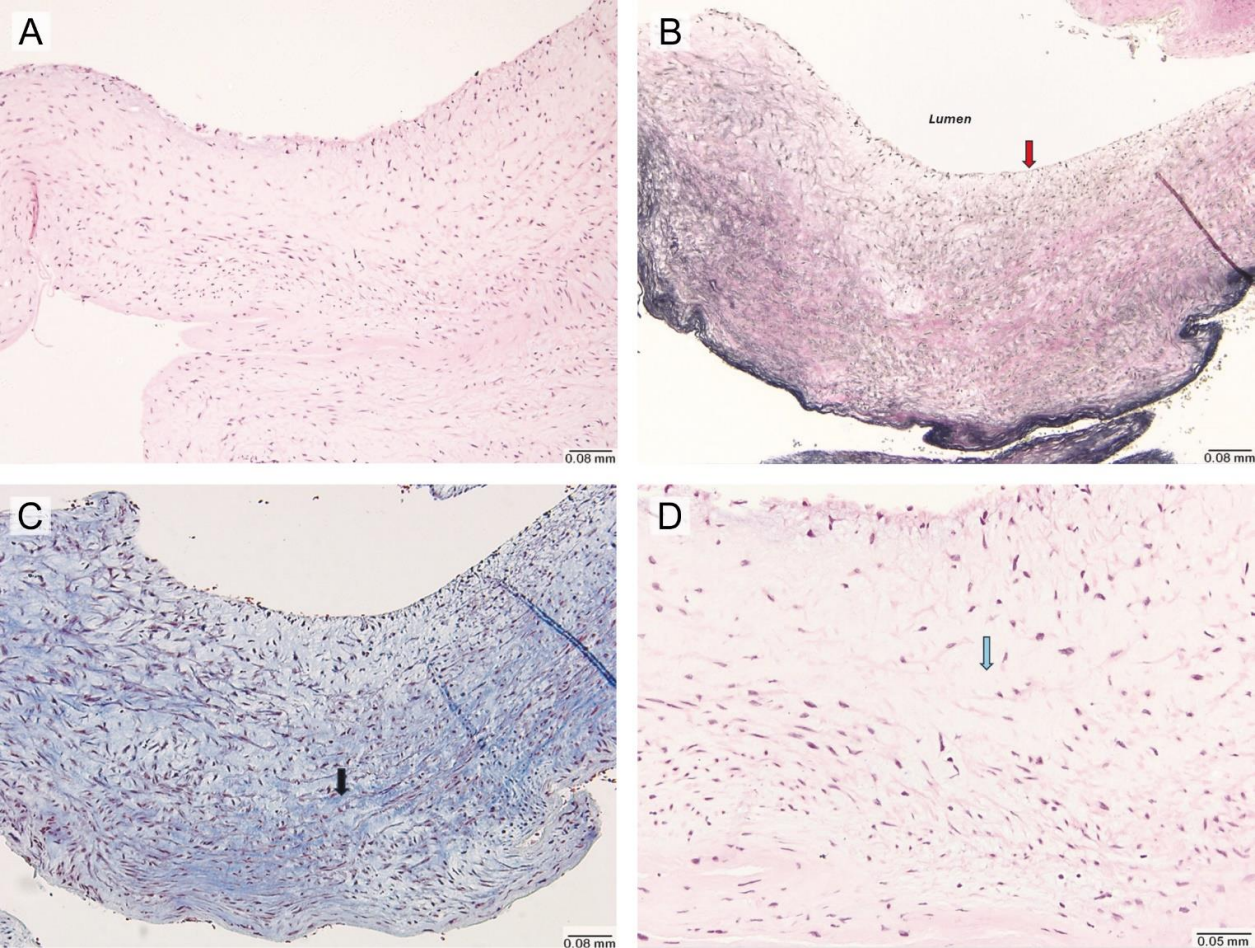
**Figure 2.** Histological findings of carotid artery web resected via endarterectomy (Hematoxylin and Eosin stain, Elastic stain). **(A)** Low-power (10×) microscopic view showing a single fragment of fibrovascular tissue, without evidence of thrombus formation, lipid deposition or inflammatory infiltrate. **(B)** Elastic stain (10×) demonstrating the absence of an internal elastic lamina (red arrow). **(C)** Trichrome stain (10×) showing increased fibrosis (black arrow). **(D)** High power (20×) view highlighting areas of myxoid degeneration (blue arrow).

A woman in her 20s without known vascular risk factors presented with dysarthria and numbness in her left hand. A non-contrast head CT scan was normal. At an outside hospital, an initial CT angiogram of the head and neck raised concern for a dissection of the right proximal internal carotid artery, and she was transferred to our institution for further evaluation. Her initial NIHSS was 1. Brain MRI showed an embolic-appearing ischemic stroke in the right frontal lobe. TTE, outpatient cardiac monitoring, and hypercoagulable workup including antiphospholipid antibody syndrome (APS) were negative. Upon reviewing the neck imaging with a neuroradiologist, the lesion was interpreted as a focal artery dissection versus a possible carotid artery web with superimposed thrombus. She was started on oral anticoagulation. Six months later, she underwent a diagnostic cerebral angiogram, which confirmed the presence of a carotid web in the proximal right internal carotid artery (Figure 1) and later right carotid endarterectomy for secondary stroke prevention. Gross examination revealed a single soft tissue fragment 1.3 x 0.5 x 0.3 cm. Examined histologic sections demonstrated fragments of fibrovascular tissue consisting of vascular smooth muscle cells in a background of myxoid degeneration, no lipid or inflammatory component was identified (Figure 2). At her most recent follow-up, 10 months after the initial event, she had not experienced a recurrent ischemic stroke.

### Case 2

**Figure 3:**
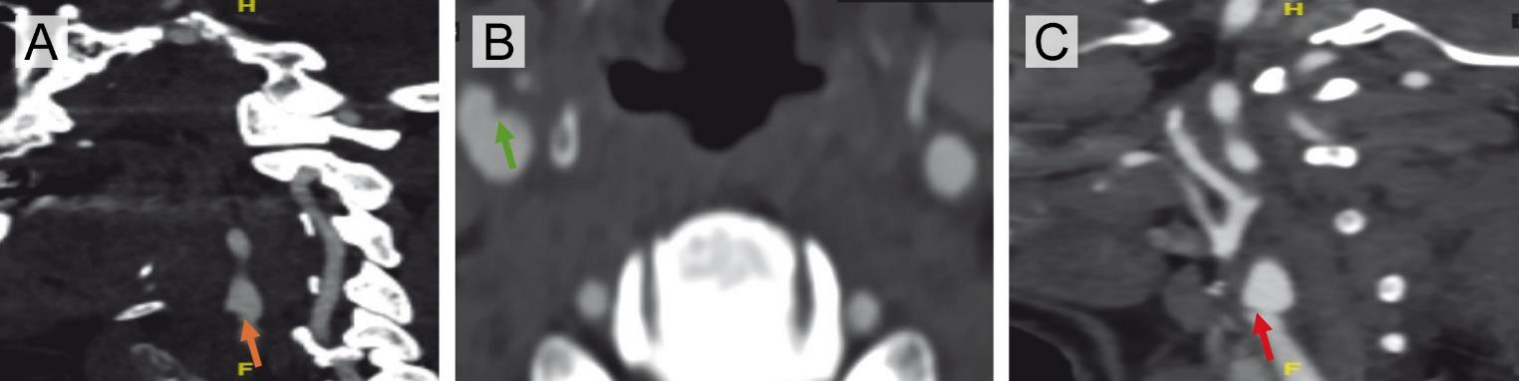
**Figure 3.** Interval development of thrombus over a carotid artery web in a patient with recurrent ischemic events. **(A)** Axial CTA at the time of large vessel occlusion demonstrates a carotid web in the right internal carotid artery with superimposed thrombus (orange arrow). **(B)** Corresponding sagittal view confirms the presence of thrombus overlying the web in the proximal right internal carotid artery (green arrow). **(C)** Imaging performed three weeks earlier during workup for TIA shows the same carotid web without thrombus (red arrow).

**Figure 4:**
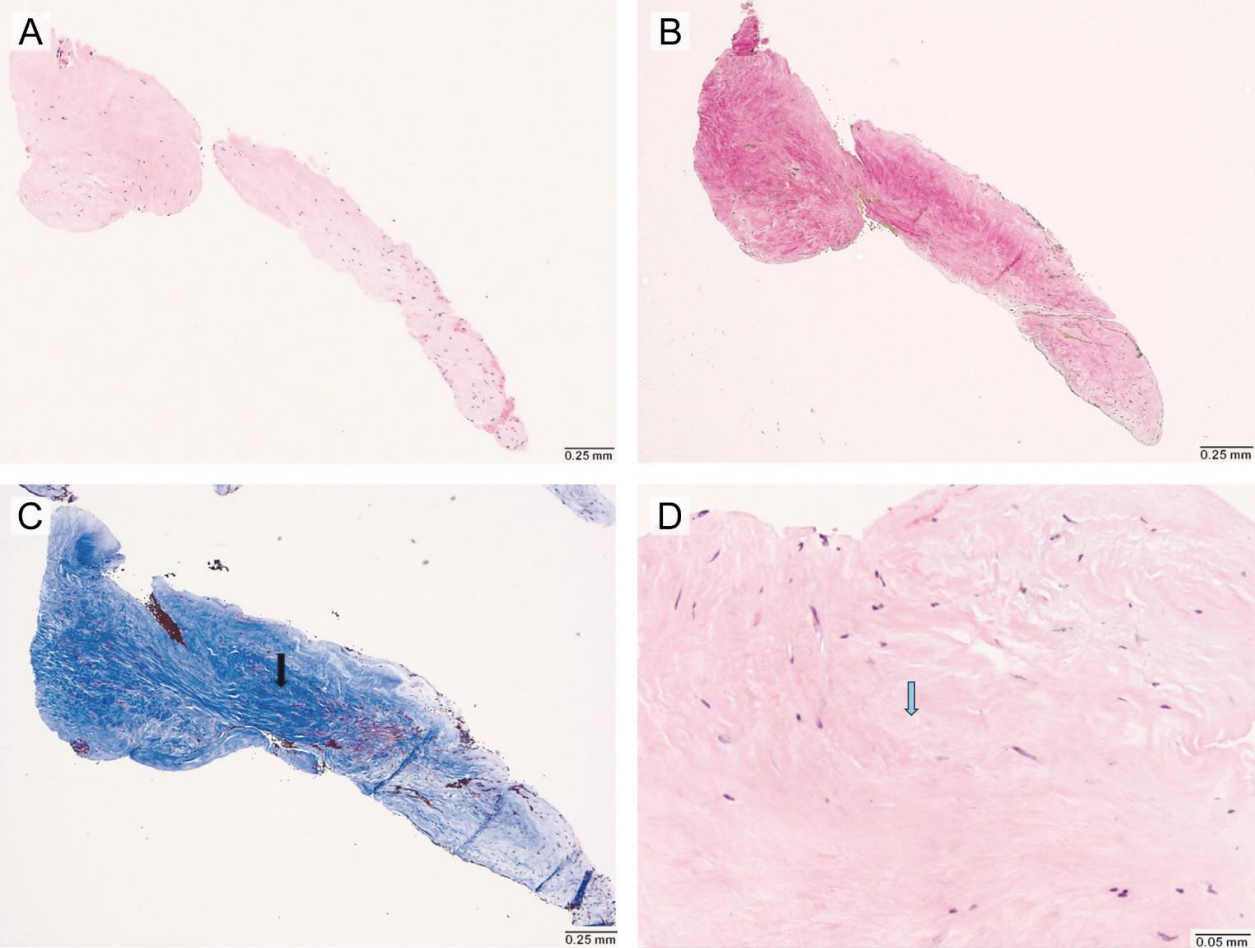
**Figure 4.** Histological findings of carotid artery web were collected via endarterectomy (Hematoxylin and Eosin stain, Elastic stain). **(A)** Low-power (4×) microscopic view showing a single fragment of fibrovascular tissue, without evidence of thrombus formation, lipid deposition or inflammatory infiltrate. **(B)** Elastic stain (4×) demonstrates the absence of staining. **(C)** Trichrome stain (4×) showing increased fibrosis (black arrow). **(D)** High power (20×) view highlighting areas of myxoid degeneration (blue arrow).

A man in his 50s with no significant past medical history presented with signs of a right middle cerebral artery (MCA) syndrome, three weeks after a transient ischemic attack (TIA) affecting the same hemisphere. His admission NIHSS was 6. A non-contrast head CT was negative for hemorrhage, and CT angiography of the head and neck revealed an occlusion of the right M1 segment. He underwent mechanical thrombectomy with successful TICI 2c recanalization. Cardiac workup, including transthoracic echocardiography (TTE), telemetry, and hypercoagulable testing, was unremarkable. Vascular imaging revealed a small right internal carotid artery (ICA) web with superimposed thrombus (Figure 2A and B), which had not been present on prior imaging performed during his initial TIA evaluation (Figure 3C). Due to recurrent vascular events ipsilateral to CW, he underwent a right carotid endarterectomy (CEA) during the same hospitalization. Gross examination of the specimen revealed multiple fragments of tan soft tissue 1.1 x 1.1 x 0.2 cm in aggregate. Examined histologic sections demonstrated fragments of fibrovascular tissue consisting of vascular smooth muscle cells in a background of myxoid degeneration and increased fibrosis. In this case, no lipid or inflammatory component was identified either (Figure 4). At his most recent follow-up, 11 months after the initial event, he had not experienced a recurrent ischemic stroke.

### Case 3

**Figure 5:**
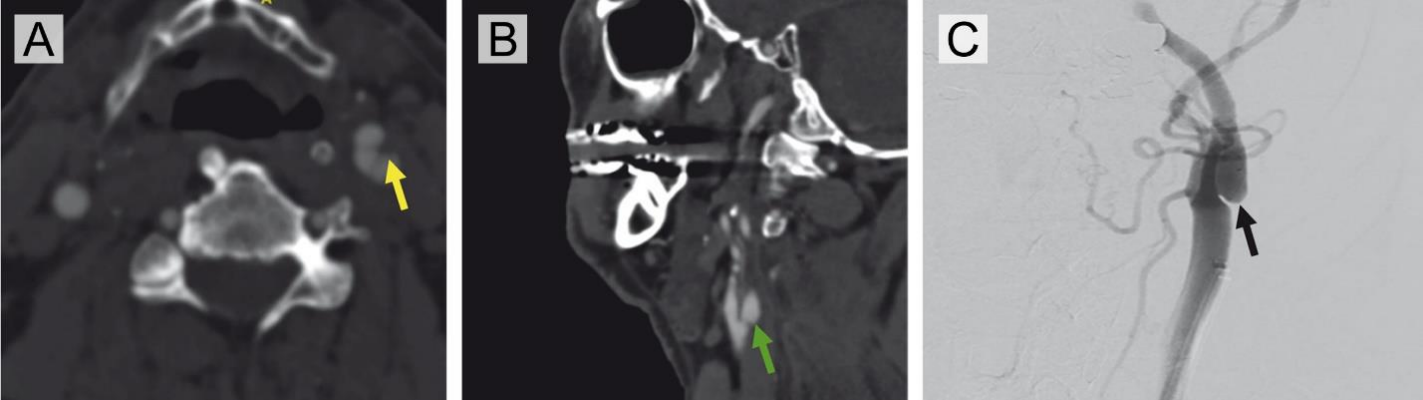
**Figure 5.** Radiographic imaging of a carotid artery web (CW) without evidence of superimposed thrombus in a patient with recurrent large-vessel occlusions (Case 3). **(A)** Axial CTA demonstrates a shelf-like projection along the posterior wall of the left internal carotid artery, consistent with a carotid web in the proximal left ICA (yellow arrow). **(B)** Corresponding sagittal CTA confirms the non-stenotic web morphology in the absence of atherosclerosis (green arrow). **(C)** Digital subtraction angiography (left common carotid contrast injection) reveals a subtle web-like filling defect in the proximal consistent with a carotid artery web (black arrow).

**Figure 6:**
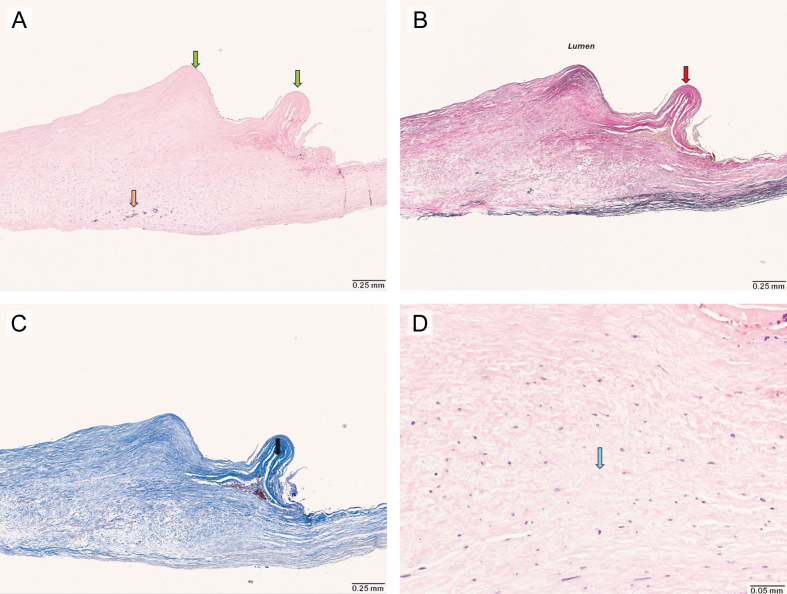
**Figure 6.** Histological findings of carotid artery web resected via endarterectomy (Hematoxylin and Eosin stain, Elastic stain, Trichrome stain). **(A)** Low-power (4×) microscopic view showing a single fragment of fibrovascular tissue, with shelf-like projections into the luminal aspect of the specimen (green arrows). There are focal calcifications (orange arrow), however there is no evidence of thrombus formation, lipid deposition or inflammatory infiltrate. **(B)** Elastic stain (4×) demonstrating the absence of an internal elastic lamina (red arrow). **(C)** Trichrome stain (4×) showing increased fibrosis in these shelf-like areas (black arrow). **(D)** High power (20×) view highlighting areas of myxoid degeneration (blue arrow).

A man in his 60s with a history of dyslipidemia presented with a left middle cerebral artery (MCA) syndrome. His NIHSS score was 14. A non-contrast head CT was negative for hemorrhage or a large ischemic core. Given that his last known well was within 4.5 hours of symptoms onset, he received intravenous thrombolytic (Tenecteplase). CT angiography of the head and neck revealed an occlusion of the M1 segment of the left MCA with proximal carotid artery web (Figure 5), and he underwent mechanical thrombectomy with successful TICI 3 recanalization. On hospital day 2, he developed worsening right arm weakness and aphasia. Imaging revealed a new proximal left M2 occlusion, and he underwent a repeat mechanical thrombectomy with TICI 2c recanalization. Further stroke workup, including TTE and age-appropriate cancer screening, was negative. During the same hospitalization, he underwent resection of a left internal carotid artery (ICA) web without stroke recurrence of TIA or ischemic stroke until his last follow up (4 months). Gross examination of the specimen revealed a 2.3 x 1.0 x 0.9 cm portion of tan tissue. Examined histologic sections demonstrated fibrovascular tissue with intimal fibroid hyperplasia, focal calcifications, and myxoid degeneration. Like the other cases, no lipid or inflammatory component was identified (Figure 6).

## Discussion

Histopathologic examination of the carotid endarterectomy specimens in this series consistently revealed fibrovascular tissue composed of vascular smooth muscle cells, intimal fibroid hyperplasia, and areas of myxoid degeneration^10^. These findings support the hypothesis that carotid webs represent an intimal variant of fibromuscular dysplasia rather than an atherosclerotic process. Notably, none of the cases demonstrated a lipid core or significant inflammatory infiltrate, distinguishing these lesions from classic atheromatous plaques^11^. The presence of myxoid degeneration and intimal hyperplasia may contribute to a prothrombotic milieu by disrupting local flow dynamics, consistent with prior imaging studies showing flow stasis and turbulence in the region of the web^12^.

The presence of superimposed thrombus was observed in multiple cases and played a critical role in diagnostic uncertainty. In acute clinical settings, thrombus layering on a carotid web may mimic the imaging appearance of soft plaque or a focal dissection, particularly when evaluated without high-resolution imaging or expert review. This can lead to initial misclassification, as illustrated by the first case in which the lesion was initially suspected to be a dissection. Advanced imaging modalities can aid in more accurate differentiation. High-resolution magnetic resonance vessel wall imaging (MR VWI) may provide indirect evidence of myxoid degeneration and help distinguish non-atherosclerotic lesions from lipid-rich plaques^13^. Additionally, intravascular optical coherence tomography (OCT) offers high-resolution cross-sectional visualization of the intimal surface and has been shown to reliably differentiate carotid webs from both soft atheromatous plaque and dissection flaps^14^. Incorporating these advanced imaging techniques can enhance diagnostic accuracy and guide appropriate management decisions in ambiguous cases.

Revascularization of the affected carotid artery through endarterectomy appears to be an effective strategy in selected patients, particularly those with recurrent ischemic events and no identifiable alternative stroke mechanism. In all three cases presented, surgical intervention was pursued after recurrence or persistent thromboembolic risk was established, with no recurrent stroke reported during follow-up. These findings are consistent with a recent meta-analysis, which demonstrated significantly reduced rates of recurrent ischemic stroke in patients with carotid artery webs who underwent carotid revascularization compared to those treated with medical management alone^9^. Despite this emerging evidence, clinical practice remains highly variable. A nationwide survey comparing vascular neurologists and neurointerventionalists found that neurointerventionalists were substantially more likely to favor revascularization, even after a first-ever ischemic stroke, whereas vascular neurologists tended to recommend continued medical therapy^15^. This variability highlights the absence of randomized controlled trials and consensus guidelines, reinforcing the need for prospective studies to establish standardized treatment pathways and better define which patients are most likely to benefit from intervention.

## Conclusion

Carotid artery webs represent a distinct, non-atherosclerotic cause of ischemic stroke, characterized histologically by intimal fibroid hyperplasia and myxoid degeneration without lipid or inflammatory components. The presence of superimposed thrombus can complicate imaging interpretation, often mimicking soft plaque or dissection. In selected patients, particularly those with recurrent ischemic events and no other identifiable etiology, carotid revascularization appears to be an effective preventive strategy. While emerging evidence, including histopathological findings and meta-analytic data, supports intervention, significant variability in clinical practice persists. Prospective, multicenter studies are urgently needed to clarify patient selection criteria and establish standardized guidelines for the management of carotid artery webs.

## Limitations

This study has several limitations due to its retrospective observational design. First, the small sample size and single-center setting may limit the generalizability of our findings. Second, carotid web diagnosis was based on imaging review by subspecialists, which, while thorough, may still be subject to interobserver variability, especially in cases with superimposed thrombus. Although we included histopathological confirmation, not all patients with suspected carotid webs undergo surgical resection, potentially introducing selection bias toward more severe or recurrent cases. Additionally, the absence of a control group receiving medical management alone precludes direct comparison of outcomes between treatment strategies. Finally, long-term follow-up was limited to less than one year.

## Conflict of interest statement

The authors declare no competing interests.

## Author contributions

F.K. drafted the manuscript. A.N. co-drafted, revised the manuscript, and prepared the histological slides. S.L., J.D., and S.Y. critically revised the manuscript.

## References

[R1] Brinster CJ, Vidal G, Bazan H, et al. Carotid Web Is an Under-recognized and Devastating Cause of Ischemic Stroke. Journal of Vascular Surgery 2021;73(3):43. 10.1016/j.jvs.2020.12.032

[R2] Guglielmi V, Compagne KCJ, Sarrami AH, et al. Assessment of Recurrent Stroke Risk in Patients With a Carotid Web. JAMA Neurol 2021;78(7):826–833. 10.1001/jamaneurol.2021.1101 PMC8111564 33970205

[R3] Choi PMC, Singh D, Trivedi A, et al. Carotid Webs and Recurrent Ischemic Strokes in the Era of CT Angiography. AJNR Am J Neuroradiol 2015;36(11):2134–2139. 10.3174/ajnr.A4431 PMC7964886 26228877

[R4] El Sayed R, Lucas CJ, Cebull HL, et al. Subjects with carotid webs demonstrate pro-thrombotic hemodynamics compared to subjects with carotid atherosclerosis. Sci Rep 2024;14:10092. 10.1038/s41598-024-60666-7 PMC11066020 38698141

[R5] Khan F, Kala N, Chang K, et al. In-hospital recurrent stroke in ipsilateral carotid web patients undergoing thrombectomy. Ann Clin Transl Neurol 2024;11:2450–6. 10.1002/acn3.52161 PMC11537123 39215397

[R6] Choi PM, Singh D, Trivedi A, et al. Carotid Webs and Recurrent Ischemic Strokes in the Era of CT Angiography. AJNR Am J Neuroradiol 2015;36(11):2134–9. 10.3174/ajnr.A4431 PMC7964886 26228877

[R7] Hart RG, Sharma M, Mundl H, et al. Rivaroxaban for Stroke Prevention after Embolic Stroke of Undetermined Source. New England Journal of Medicine 2018;378(23):2191–2201. 10.1056/NEJMoa1802686 29766772

[R8] Dabigatran for Prevention of Stroke after Embolic Stroke of Undetermined Source. N Engl J Med 2019;380:1906-1917. 10.1056/NEJMoa1813959 31091372

[R9] Khan F, Gujjar AR, Fletcher L, et al. Carotid Revascularization vs. Medical Management for Ischemic Stroke with Ipsilateral Carotid Web: A Systematic Review and Meta-Analysis (S25.005). Neurology 2025;104(7_Supplement_1):2690. 10.1212/WNL.0000000000210516 40503762

[R10] Wang LZ, Calvet D, Julia P, et al. Is carotid web an arterial wall dysplasia? A histological series. Cardiovasc Pathol 2023;66:107544. 10.1016/j.carpath.2023.107544 37263518

[R11] Stary HC, Chandler AB, Dinsmore RE, et al. A definition of advanced types of atherosclerotic lesions and a histological classification of atherosclerosis. A report from the Committee on Vascular Lesions of the Council on Arteriosclerosis, American Heart Association. Circulation 1995;92(5):1355–1374. 10.1161/01.CIR.92.5.1355 7648691

[R12] Compagne KCJ, Dilba K, Postema EJ, et al. Flow Patterns in Carotid Webs: A Patient-Based Computational Fluid Dynamics Study. AJNR Am J Neuroradiol 2019;40(4):703–708. 10.3174/ajnr.A6012 PMC7048501 30872422

[R13] Su Y, Stein LA, Cucchiara BL, Song JW. Vessel Wall Imaging of a Carotid Web. Annals of Neurology 2022;92(2):335–336. 10.1002/ana.26424 35638145

[R14] Al-Bayati AR, Nogueira RG, Sachdeva R, et al. Optical Coherence Tomography in the Evaluation of Suspected Carotid Webs. J NeuroIntervent Surg 2023;jnis-2023-020813. 10.1136/jnis-2023-020813 38041658

[R15] Khan F, Mallick D, Wolman D, et al. Management of carotid artery web: a nationwide survey of vascular neurologists versus neurointerventionalists [Internet]. Journal of NeuroInterventional Surgery 2025;[cited 2025 May 21 ] Available from: https://jnis.bmj.com/content/early/2025/05/16/jnis-2025-023232. 10.1136/jnis-2025-023232 40379476

